# Towards the Laboratory Maintenance of *Haemagogus janthinomys* (Dyar, 1921), the Major Neotropical Vector of Sylvatic Yellow Fever

**DOI:** 10.3390/v15010045

**Published:** 2022-12-23

**Authors:** Adam Hendy, Nelson Ferreira Fé, Danielle Valério, Eduardo Hernandez-Acosta, Bárbara A. Chaves, Luís Felipe Alho da Silva, Rosa Amélia Gonçalves Santana, Andréia da Costa Paz, Matheus Mickael Mota Soares, Flamarion Prado Assunção, José Tenaçol Andes, Chiara Andolina, Vera Margarete Scarpassa, Marcus Vinícius Guimarães de Lacerda, Kathryn A. Hanley, Nikos Vasilakis

**Affiliations:** 1 Department of Pathology, University of Texas Medical Branch, 301 University Blvd, Galveston, TX 77555-0609, USA; 2 Centro de Entomologia, Fundação de Medicina Tropical Doutor Heitor Vieira Dourado (FMT-HVD), Manaus 69040-000, AM, Brazil; 3 Instituto de Pesquisa Clínica Carlos Borborema (IPCCB), Fundação de Medicina Tropical Doutor Heitor Vieira Dourado (FMT-HVD), Manaus 69040-000, AM, Brazil; 4 Department of Biology, New Mexico State University, Las Cruces, NM 88003-8801, USA; 5 Graduate Program in Entomology, Instituto Nacional de Pesquisas da Amazônia, Manaus 69067-375, AM, Brazil; 6 Department of Medical Microbiology, Radboud University Nijmegen Medical Centre, 6525 GA Nijmegen, The Netherlands; 7 Coordenação de Biodiversidade, Instituto Nacional de Pesquisas da Amazônia, Manaus 69067-375, AM, Brazil; 8 Instituto Leônidas and Maria Deane, Fiocruz Amazonas, Manaus 69057-070, AM, Brazil; 9 Center for Biodefense and Emerging Infectious Diseases, University of Texas Medical Branch, 301 University Blvd, Galveston, TX 77555-0609, USA; 10 Institute for Human Infection and Immunity, University of Texas Medical Branch, 301 University Blvd, Galveston, TX 77555-0609, USA; 11 Center for Tropical Diseases, University of Texas Medical Branch, 301 University Blvd, Galveston, TX 77555-0609, USA; 12 Center for Vector-Borne and Zoonotic Diseases, University of Texas Medical Branch, 301 University Blvd, Galveston, TX 77555-0609, USA

**Keywords:** *Haemagogus janthinomys*, sylvatic, arbovirus, yellow fever, Flaviviridae, *Flavivirus*, blood feeding, forced mating

## Abstract

*Haemagogus (Haemagogus) janthinomys* (Dyar, 1921), the major neotropical vector of sylvatic yellow fever virus, is notoriously difficult to maintain in captivity. It has never been reared beyond an F_1_ generation, and almost no experimental transmission studies have been performed with this species since the 1940s. Herein we describe installment hatching, artificial blood feeding, and forced-mating techniques that enabled us to produce small numbers of F_3_ generation *Hg. janthinomys* eggs for the first time. A total of 62.8% (1562/2486) F_1_ generation eggs hatched during ≤10 four-day cycles of immersion in a bamboo leaf infusion followed by partial drying. Hatching decreased to 20.1% (190/944) in the F_2_ generation for eggs laid by mosquitoes copulated by forced mating. More than 85% (79/92) female F_2_ mosquitoes fed on an artificial blood feeding system. While we were unable to maintain a laboratory colony of *Hg. janthinomys* past the F_3_ generation, our methods provide a foundation for experimental transmission studies with this species in a laboratory setting, a critical capacity in a region with hyper-endemic transmission of dengue, Zika, and chikungunya viruses, all posing a risk of spillback into a sylvatic cycle.

## 1. Introduction

*Haemagogus (Haemagogus) janthinomys* (Dyar, 1921) was first implicated in the natural transmission of sylvatic yellow fever virus (YFV, genus *Flavivirus*, family *Flaviviridae*) in the Americas in 1938, when it was isolated from pools of forest mosquitoes collected in Rio de Janeiro State, Brazil [1,2]. Alongside *Hg.* (*Conopostegus*) *leucocelaenus* (Dyar and Shannon, 1924), *Hg. janthinomys* is considered the major vector of YFV [3,4] and is also thought to be a prominent vector of Mayaro virus (MAYV, genus *Alphavirus*, family *Togaviridae*), in neotropical forests [5,6]. Despite its obvious medical importance, almost no experimental transmission studies have been performed with this species since the early studies of YFV in the 1930s and 40s [7,8]. The lack of research is often attributed to difficulties rearing these mosquitoes under laboratory conditions [9,10,11], although there has been little effort to overcome this problem in recent years.

Early attempts to keep *Hg. janthinomys* alive in cages beyond the extrinsic incubation period of YFV; around 14 days or more for this species, proved difficult [12,13]. To convincingly demonstrate vector competence [10,14], it was necessary to maintain adult female mosquitoes individually in small glass vials containing a layer of damp cotton and a disc of filter paper [10,15]. These were either field collected mosquitoes or the F_1_ progeny of field collected mosquitoes that were blood fed on monkeys or marsupials in captivity [8,10,15]. Specimens were kept alive for a maximum of around 50 days [15], which was ample time to demonstrate onward transmission of the virus to uninfected hosts [10]. Since then, a modified vial-based method has been shown to increase adult longevity up to 72 days and improve upon egg-yields documented in earlier studies [16]. Despite this progress, there have been no published vector competence studies involving *Hg. janthinomys* since 1956 [9]. Dengue, Zika (DENV, ZIKV, both genus *Flavivirus*), and chikungunya viruses (CHIKV, genus *Alphavirus*) have all emerged or resurged in the Americas during this time [17]. They are currently only known to circulate in human endemic cycles in the region, involving *Aedes* (*Stegomyia*) *aegypti* (Linnaeus, 1762) and *Ae.* (*Stg.*) *albopictus* (Skuse, 1894) mosquitoes. However, the presence of susceptible sylvatic hosts and competent vectors creates risk for virus spillback and the emergence of novel sylvatic cycles [18,19], not amenable to control interventions, that would be a source of spillover akin to YFV.

*Haemagogus janthinomys* mosquitoes possess several physiological and behavioral traits that make laboratory maintenance problematic. They lay their eggs on the edges of transient water bodies in tree holes and bamboo [2,20,21]. Consequently, they show some drought tolerance and hatch in installments as a survival mechanism [22,23]. Eclosion requires multiple cycles of immersion and drying, making it difficult to obtain high numbers of larvae on demand. While a slow process, problems with installment hatching can partly be overcome through perseverance [23,24], and F_1_ adults reared from field collected mosquitoes have been used to perform fairly extensive survival and vector competence studies [15]. However, laboratory maintenance of *Hg. janthinomys* has never progressed beyond the F_1_ generation. These mosquitoes are reluctant to mate in cages [25], and attempts to encourage mating have either been unsuccessful [9,15,25] or considered too difficult [11,26,27].

The absence of a simple blood feeding technique further complicates the task of colonizing *Hg. janthinomys*. Blood-soaked cotton pledgets and membrane feeders have been used to deliver virus-infected blood to *Hg.* (*Hag.*) *equinus* (Theobald, 1903) [28] and *Hg. leucocelaenus* [28], while mice and human blood have been used to maintain *Hg. equinus* in colony [29]. It is unclear whether *Hg. janthinomys* will feed readily on adult mice, but they will bite baby mice, as these have previously been used in vector competence studies [7,10]. Other blood sources utilized in experimental work involving *Hg. janthinomys* have included rhesus macaques [9,13,30], various species of neotropical monkeys [7,8,10,31], marsupials [8], and YFV vaccinated human volunteers [16], none of which are suitable for long-term colony maintenance.

Soon after 1956, when the last published *Hg. janthinomys* vector competence studies involving just seven mosquitoes were abandoned due to a lack of material [9], techniques were developed to induce copulation between aedine mosquitoes that rarely mate under laboratory conditions [32]. Forced mating is now mainly used to colonize eurygamous malaria vectors reluctant to mate in cages [33,34,35], but has never been applied to *Hg. janthinomys*. In this study, we describe our progress towards the laboratory maintenance of *Hg. janthinomys*, in which we used artificial blood feeding and forced mating techniques to produce small numbers of F_3_ generation eggs.

## 2. Materials and Methods

### 2.1. Ethics

Field collections of *Hg. janthinomys* differed from human landing collections in that mosquitoes were captured using nets prior to landing and no skin was deliberately exposed to attract mosquitoes. All collectors were vaccinated against YFV and wore trousers and either a long-sleeved shirt or repellent to minimize the risk of being bitten. Mosquito collections were approved by local environmental authorities (SISBIO license 57003-6) and the study did not involve endangered or protected species. Artificial feeding methods involving human blood were reviewed and approved by the Fundação de Medicina Tropical Doutor Heitor Vieira Dourado (FMT-HVD) Ethics Review Board (CAAE: 53963521.0.0000.0005).

### 2.2. Field Collected Mosquitoes

#### 2.2.1. Source Material

Host-seeking female *Haemagogus* species mosquitoes were collected at the Adolpho Ducke forest reserve bordering Manaus in the Brazilian Amazon between February and April 2022. Approaching mosquitoes were collected with handheld nets at ground level and on elevated platforms during the daytime (10:00–15:00) at a treefall (02.92537° S, 059.96582° W) [36]. Captured mosquitoes were transferred to a 16 × 19.5 cm cylindrical cardboard cage and provided with cotton wool soaked with mineral water for hydration. The cage was placed in a Styrofoam box in the shade throughout the day to prevent mosquito desiccation. All subsequent work took place in an air-conditioned insectary at FMT-HVD at 26 °C (±1 °C) and 70–80% relative humidity unless stated. The insectary was maintained in a constant 12 h light-dark cycle with changes in photoperiod occurring at 06:00 and 18:00 daily.

#### 2.2.2. Blood Feeding

Immediately upon returning from the field, mosquitoes were transferred to a 15 × 15 cm plastic cage covered with fine mesh netting (Figure S1) and were supplied with two cotton wool balls, one soaked with distilled water and the other a 10% sugar solution. Mosquitoes were left to settle until the following evening when the water and sugar solution were removed. Feeding was then attempted the next morning at around 08:00. Starving *Hg. janthinomys* for such long periods prior to blood feeding resulted in mortality of up to 41.8% on several occasions (Dataset S1).

**Figure 1:**
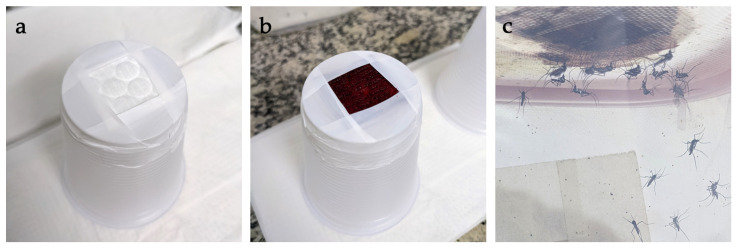
Artificial blood feeding. (**a**) cotton pad secured to the base of a plastic feeding cup with Teflon tape; (**b**) feeding cup after the application of blood; and (**c**) *Hg. janthinomys* feeding on a cup inverted over the top of a cage and partially filled with 45 °C water.

Human blood was drawn from healthy volunteers into 9 mL sodium heparin tubes (Greiner Bio-One, São Paulo, Brazil) by a trained technician. Blood was either used fresh or was refrigerated for ≤four days at 4 °C and heated to 37 °C in a water bath for 15–20 min before use. Feeders were prepared using round cotton pads and 180 mL disposable plastic drinking cups. Cotton pads were cut into approximately 2 × 2 cm squares and secured to the base of the cups using Teflon tape (Tecnoflon-Brasfita, São Paulo, Brazil) (Figure 1a). About 2 mL blood was applied to each pad until saturated (Figure 1b) and feeders were inverted over the top of the cage (Figure 1c). The blood was kept warm by adding approximately 100 mL of 45 °C water to the plastic cups. The water in each cup was changed once or twice when the temperature decreased below 36 °C, resulting in around 60 min feeding time. Field collected mosquitoes were offered blood on a single occasion. Mosquitoes were individually aspirated and those that had fully or partially engorged were separated into a 15 × 15 cm plastic cage and left to rest with water and sugar solution for five days before oviposition was attempted. Those that did not feed were discarded.

#### 2.2.3. Oviposition

At five days post blood feeding, groups of up to 10 gravid mosquitoes were aspirated into 50 mL Falcon tubes where they were anesthetized by exposure to ethyl acetate vapor. To achieve this, a scrap of paper towel was twisted, dipped in ethyl acetate, and then secured inside a tube near its lid, ensuring the mosquitoes did not make contact. Since *Hg. janthinomys* are phototactic, this was possible by directing the opposite end of the tube towards natural light. This also prevented mosquitoes escaping when manipulating the lid. Once fully relaxed (usually less than 60 s), mosquitoes were placed in a Petri dish, and each had a wing removed under a stereomicroscope. They were then transferred either individually or in groups of up to nine mosquitoes into Petri dishes lined with 80 g filter paper (J Prolab, São José dos Pinhais, Brazil), placed above a damp cotton pad to maintain humidity (Figure S2). Mosquitoes were left for at least three days to oviposit before F_1_ eggs were recovered and hatching was attempted. Additional drops of water were added to filter papers as required during this time.

### 2.3. F_1_ Generation

#### 2.3.1. Installment Hatching and Rearing

Egg papers were immersed as soon as possible in 2.5 L white plastic rearing trays containing approximately 500 mL 1:1 bamboo leaf infusion to distilled water. The leaf infusion was prepared two days in advance in a 5 L clear plastic bottle containing approximately 60 g fresh bamboo (*Phyllostachys* sp.) leaves, collected on FMT-HVD grounds, and distilled water. The bottle opening was covered with netting to prevent insects from entering and the infusion was kept at room temperature in an outdoor storage building. Since *Hg. janthinomys* hatch in installments [22], egg papers were generally submerged for two days and then partially dried for two days for up to ten immersion cycles. Partial drying involved laying egg papers on damp paper towels. It was critical that egg papers did not fully dry as eggs would soon collapse. To combat mold growth, body parts of dead mosquitoes were removed before the first immersion. Additionally, egg papers were placed in a weak bleach solution (1:3 2.5% NaClO to distilled water) for approximately 10 s after the second or third immersion cycle, or whenever mold growth was observed, then rinsed for one minute in a tray of distilled water. Larvae were reared to pupae in the water in which they emerged and a small amount of Fleischmann instant dry yeast (AB Brasil Industria e Comércio de Alimentos Ltda., São Paulo, Brazil) was added if required to increase the size of adults. This improved chances of survival and larger adults were easier to manipulate when attempting copulation (Section 2.3.2). Rearing trays were checked daily for rapid biofilm growth which, if present, was removed by dragging a paper towel smoothly across the surface of the water. Pupae were transferred to glass tumblers containing 150 mL distilled water placed inside 15 × 15 cm plastic cages (Section 2.2.2) until adults emerged. The number of days until emergence of the first larva, pupa, and adult was recorded in each tray as an indicator of development time. Adult mosquitoes were then separated by date of emergence, usually within a range of 1–2 days, into new 15 × 15 cm cages until required for copulation.

#### 2.3.2. Copulation by Forced Mating

Forced mating of *Hg. janthinomys* was based on techniques applied to eurygamous *Anopheles* species [33]. We aimed to use approximately 3–5-day old adult males and 1–3-day old females, although no material was wasted, regardless of age. The work took place in a 26 °C air-conditioned room at the FMT-HVD Center for Entomology.

**Figure 2:**
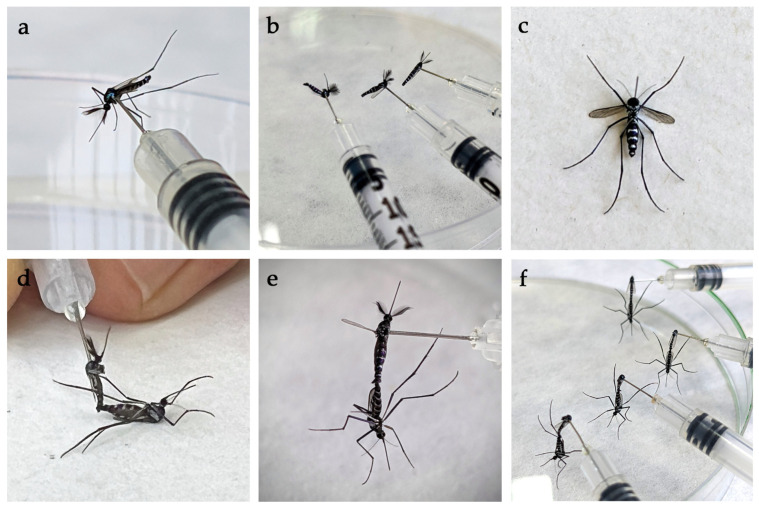
Forced mating F_1_ generation *Hg. janthinomys*. (**a**) a male mosquito mounted laterally through the thorax on a 0.5 mL syringe; (**b**) mounted males with legs removed resting on a Petri dish; (**c**) a fully anesthetized female with legs relaxed; (**d**) introduction of mosquito terminalia at an angle of about 90°; (**e**) fully interlocked male and female; and (**f**) decapitated male and female mating pairs at rest on the lid of a Petri dish.

Male and non-blood fed female mosquitoes were anesthetized by exposure to ethyl acetate vapor as described in Section 2.2.3. Initially, 5–6 males were mounted laterally through the thorax on needles of 0.5 mL insulin syringes (Figure 2a). Mosquito legs were removed using soft entomological forceps (Figure 2b) but males were not decapitated at first, which is a common technique to induce mating [33,34]. However, when mosquito numbers increased, the heads of males were removed to speed up the copulation process. Around 2–3 anesthetized females were placed, with their ventral surface facing up (Figure 2c), on the lid of a white microcentrifuge box which could be rotated to allow easy manipulation. The male and female terminalia were introduced at an angle of slightly more than 90° (Figure 2d) (Video S1). If the mosquito pairs were still unresponsive after a short time, a different male was offered to a female. Male mosquitoes were reused once or twice if they remained viable after copulation. Once the terminalia were fully interlocked (Figure 2e), mating pairs were left to rest on the lid of a Petri dish until they separated (Figure 2f). The female was then quickly, but gently, aspirated and transferred to a new pre-prepared cage with distilled water and 10% sugar solution, and left to rest until the next day. Additional mosquitoes were prepared as needed.

#### 2.3.3. Modifications to Blood Feeding and Oviposition

Only small numbers of F_1_ females blood fed initially and repeatedly separating blood fed from non-blood fed females resulted in high mortality. To counter this, all copulated F_1_ females, regardless of the strength of interaction with the male, were separated into two 20 × 18 cm plastic cages containing oviposition substrates. The substrates comprised 150 mL clear plastic containers lined with filter paper and a thick layer of cotton wool saturated with distilled water (Figure S3). An extra layer of filter paper was placed above the cotton wool to prevent mosquitoes becoming entangled. Mosquitoes were offered blood daily following the method described in Section 2.2.2, in the hope that some would refeed and survive through multiple gonotrophic cycles. Water and sugar solution were removed no more than two hours before feeding to reduce the risk of desiccation. Oviposition substrates were usually changed every seven days.

### 2.4. F_2_ Generation

#### Similarities with Previous Generations and Further Modifications

F_2_ generation eggs were immersed, hatched, and reared in a bamboo leaf infusion for up to nine cycles as described in Section 2.3.1. To overcome low egg yields, we reverted to separating blood fed from non-blood fed females using a slightly modified method, and only used blood fed mosquitoes for copulation. Adult males were separated into 15 × 15 cm cages by date of eclosion. Females were added to a single 15 × 15 cm modified feeding cage covered with fine mesh netting and with a lateral hole plugged with cotton wool. Female mosquitoes were offered blood daily as described in Section 2.2.2. However, the 45 °C water was not changed, and blood was only offered for 20–30 min. Mosquitoes that had visibly fed were aspirated via the lateral hole. Blood fed females were copulated and left to rest for at least five days before oviposition was attempted using individual mosquitoes placed in filter-paper lined Petri dishes (Section 2.2.3).

### 2.5. Dissections and Hind Leg Measurements

Once dead, a subsample of individually maintained F_2_ generation mosquitoes were dissected in a drop of saline solution using a Zeiss Stemi SV 6 stereomicroscope and fine needles. The presence/absence of blood and eggs in the abdomen, and the number of eggs retained, was recorded. Additionally, a 10 mm stage micrometer with 0.1 mm divisions (Ted Pella Inc., Redding, CA, USA) was used to measure the length of the hindtarsi of a subsample of field collected, F_1_, and F_2_ generation mosquitoes as a proxy for relative size.

### 2.6. Identifying Mosquitoes

Field collected mosquitoes were sampled at a site where >97% of anthropophilic *Haemagogus* were known to be *Hg. janthinomys* [36]. All individually maintained mosquitoes (field collected and F_2_ adult females) were morphologically identified using a stereomicroscope and relevant taxonomic keys [2,37,38,39].

### 2.7. Statistics

Wilcoxon rank sum tests for non-normally distributed data were used to compare egg to larva, larva to pupa, and pupa to adult development times between generations. A simple linear regression was used to investigate the effect of larval density on development time. A Fisher’s exact test was used to compare the number of individually maintained mosquitoes ovipositing between field collected and F_2_ generations. Wilcoxon rank sum tests were also used to compare the number of days until the first eggs were laid, and the number of eggs laid between respective generations. A Kruskal–Wallis test and pairwise post hoc Wilcoxon Each Pair comparisons were used to compare the length of hindtarsi of adult mosquitoes between generations. Analyses were performed using JMP 14 [40].

## 3. Results

### 3.1. Source Material

A total of 418 adult female *Haemagogus* spp. were collected at the Ducke reserve between February and April 2022, of which 226 (54.1%) blood fed at first attempt in the laboratory (Table 1). Eggs that were morphologically distinct from *Hg. janthinomys* were removed from egg papers before hatching was attempted. Small numbers of *Hg. leucocelaenus* adults were also removed from cages of F_1_ mosquitoes. The remaining individually maintained adults were morphologically identified as *Hg. janthinomys*.

### 3.2. Oviposition of Field Collected and F_1_ Generation Mosquitoes

The 226-blood fed, field collected adults produced 2486 F_1_ eggs (11 per mosquito), which were laid in Petri dishes and subsequently used for hatching. Since few F_1_ adults blood fed initially, and repeated manipulation resulted in mortality, we added 445 copulated F_1_ females to cages containing oviposition substrates as described in Section 2.3.3. These produced 944 F_2_ eggs (2.1 per mosquito) over 55 days (38.0% of the original 2486).

### 3.3. Installment Hatching of F_1_ and F_2_ Generation Eggs

**Figure 3:**
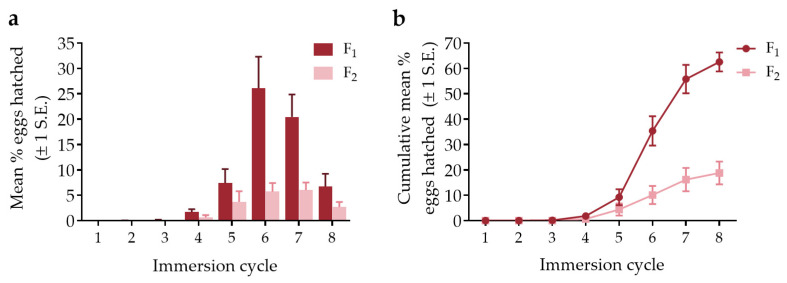
*Haemagogus janthinomys* egg eclosion shown for the first eight immersion cycles for N = 10 F_1_ and N = 8 F_2_ egg papers (total 2486 F_1_ and 944 F_2_ eggs, respectively). Eggs were immersed as soon as possible once recovered from Petri dishes or cages. (**a**) mean percentage of eggs hatched ± 1 standard error (S.E.); and (**b**) the cumulative mean percentage of eggs hatched (±1 S.E.).

Almost no hatching occurred during the first three immersion cycles for both the F_1_ and F_2_ generations (Figure 3, Dataset S1). Eclosion began to increase during the fourth and fifth cycles and peaked during the sixth and seventh cycles for the F_1_ generation, before quickly declining. The F_2_ generation followed a similar pattern but there was no clear peak in egg hatching. The cumulative mean % eggs hatched after eight immersion cycles was considerably higher for the F_1_ generation (mean 62.5%) than the F_2_ generation (18.8%).

### 3.4. Development from Egg to Adult

**Figure 4:**
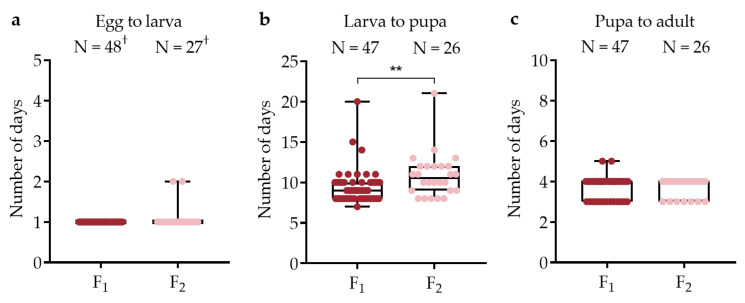
Egg to adult development times for F_1_ and F_2_ *Hg. janthinomys* in trays where at least one egg hatched. Box plots show median values and interquartile range (IQR) for development times based on the number of days until the emergence of the first (**a**) larva, (**b**) pupa, and (**c**) adult in each rearing tray. Significant differences indicated by asterisks (** *p* < 0.01). ‘N =’ = number of trays where at least one egg hatched. ^†^ All larvae died in one rearing tray in each generation.

Rearing was relatively straightforward and successful once larvae had emerged with a total 86.1% (1345/1562) F_1_ and 93.7% (178/190) F_2_ mosquitoes surviving to adults. The first larvae usually hatched within 5–6 h of immersion, although results were recorded the following day. There was no significant difference in the number of days until the first larva emerged between generations (Wilcoxon test, DF = 1, χ^2^ = 3.6, *p* = 0.058), but there was a difference in development time from first larva until first pupa (DF = 1, χ^2^ = 7.38, *p* = 0.007), with the F_2_ generation developing more slowly (Figure 4). A simple linear regression revealed a negative relationship (DF = 1, F = 6.9, *p* = 0.01) between larval density (number of eggs hatched) per container and the larval to pupal development time for both generations combined. There was no difference in development time between generations from first pupa until first adult (Wilcoxon test, DF = 1, χ^2^ = 0.04, *p* = 0.83). The development time from egg to adult was completed within a mean 15 days (min = 12, max = 26). The male to female adult sex ratio was 1:1.1 for the F_1_ generation and 1:1.2 for the for the F_2_ generation.

### 3.5. Individually Maintained Mosquitoes

**Figure 5:**
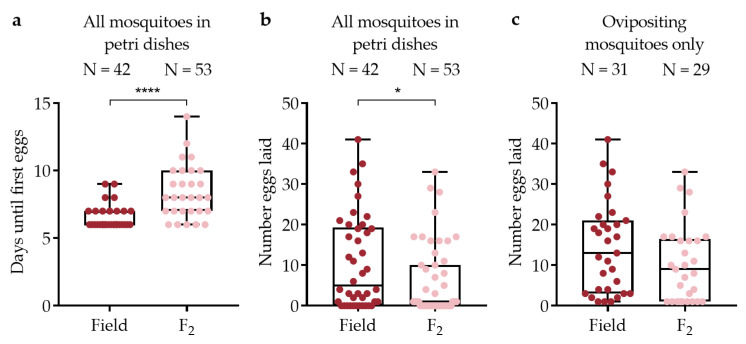
Egg development and oviposition for individually maintained field collected (Field) and F_2_ mosquitoes. Box plots show median values and IQR for (**a**) number of days until first eggs were laid; (**b**) number of eggs laid per mosquito for all mosquitoes in Petri dishes; and (**c**) number of eggs laid per mosquito for ovipositing mosquitoes in Petri dishes. Significant differences indicated by asterisks (* *p* < 0.05, **** *p* < 0.001). Number of individual mosquitoes (N) above bars.

A subset of field collected and F_2_ generation mosquitoes were maintained individually in Petri dishes to obtain detailed data about egg development and oviposition. Oviposition rates were higher among field collected mosquitoes (31/42, 73.8% laid eggs) than the F_2_ generation (29/53, 54.7%), although this difference was not significant (Fisher’s Exact test, DF = 1, N = 95, *p* = 0.086). Field collected mosquitoes laid their first eggs more quickly than F_2_ generation mosquitoes (Figure 5a, Table 2) (Wilcoxon test, DF = 1, χ^2^ = 18.4, *p* < 0.0001). The median number of eggs laid by mosquitoes added to Petri dishes that had a chance to oviposit was higher for the field collected generation than for the F_2_ generation (DF = 1, χ^2^ = 6.18, *p* = 0.013) (Figure 5b). The same was also true when considering ovipositing mosquitoes only (Figure 5c), although the difference was not significant (DF = 1, χ^2^ = 2.70, *p* = 0.10). There was considerable egg retention among all dissected F_2_ mosquitoes (N = 46, mean eggs retained ±1 S.E. = 17.0 ± 1.74) and among those that laid at least one egg (N = 29, mean eggs retained ±1 S.E. = 13.5 ± 2.09).

### 3.6. Mosquito Size by Generation

A comparison of the size of female mosquitoes based on a subsample of 269 specimens (N = 102 field, 95 F_1_, and 72 F_2_ mosquitoes) revealed a significant difference in mean hindtarsus length between generations (Kruskal–Wallis test, DF = 2, χ^2^ = 44.0, *p* < 0.0001). Post hoc Wilcoxon Each Pair tests showed that both laboratory generations were significantly larger than the field collected generation (*p* < 0.0001 for both comparisons), while the F_2_ generation was also larger than the F_1_ generation (*p* = 0.0001).

## 4. Discussion

We reared *Hg. janthinomys* beyond an F_1_ generation for the first time. While our techniques could not sustain a laboratory colony, the study provides a foundation for improving maintenance methods. Crucially, our artificial blood feeding method will enable future in situ *Hg. janthinomys* vector competence studies to be performed using F_1_ adult females, which are relatively easy to rear in large numbers. These are necessary in a region affected by hyperendemic transmission of DENV, ZIKV, and CHIKV, and where the risk of sylvatic spillback is high.

We collected relatively small numbers of mosquitoes in the field over eight weeks, which was sufficient to perform this study but not ideal for establishing a colony. Sampling more intensively over a shorter time would yield higher numbers of F_1_ mosquitoes during each immersion cycle, allowing more control when blood feeding and copulating mosquitoes of a certain age. Shorter, more intensive collections have been successfully used to establish *Anopheles* colonies elsewhere [35,41], but are even more important for *Hg. janthinomys* given the additional difficulties associated with installment hatching.

The artificial feeding method was effective in delivering blood to each generation. We discarded unengorged field collected mosquitoes after the first feeding attempt since they did not appear to feed well thereafter, and we were unable to quantify feeding rates of F_1_ generation mosquitoes maintained in cages since they could not easily be counted. However, we were able to show that more than 85% of F_2_ females fed when presented with blood daily. Anecdotally, we observed blood feeding starting at around five days post emergence. However, we did not systematically record this since the small numbers of F_2_ adults available would have needed to be maintained and fed individually by day of emergence. If confirmed, our observation would be consistent with delayed feeding responses of arboreal *Sabethes* (*Sabethoides*) *chloropterus* (von Humboldt, 1819) which do not feed before five days post emergence [42], but slower than *Hg. equinus* [28] and the major *Aedes* vectors [43,44], which all begin feeding within three days post emergence. It was necessary to heat water used in the feeding system to 45 °C to achieve good rates of blood feeding, but it was not necessary to change the water as we did with earlier generations. Most mosquitoes that were going to feed did so within 20–30 min of being offered a blood meal.

Our forced mating technique differed slightly from the WHO method for *Anopheles* mosquitoes [33], which recommends introducing the terminalia at about 45 degrees. We found that an angle of slightly more than 90 degrees was appropriate for *Hg. janthinomys*, which is similar to the angle used when forced mating *Ae. aegypti* [45]. Other factors influencing forced mating outcome may include mosquito age and duration of copulation [46,47,48]. We attempted to use 3–5-day old adult males and 1–3-day old females. However, this was difficult since the effects of installment hatching meant that we often only had small numbers of mosquitoes to work with on a given day.

Oviposition rates were higher among field collected mosquitoes than the F_2_ generation, although the difference was not significant. The number of F_2_ generation mosquitoes that laid eggs (29) was also not consistent with the number copulated and added to Petri dishes (53), which may indicate that some mosquitoes were not inseminated despite coupling. This has been reported elsewhere [46,49] and it has been stated that at least six seconds of firm copulation are needed for insemination to occur in *Ae. aegypti* [47]. Our copulations lasted from a few seconds to several minutes, but the exact duration was not recorded on this occasion. The high number of eggs retained may also be indicative of unsuccessful insemination [50]. It was always likely that forced mating would be less effective than the natural process and it would be beneficial to compare insemination rates between generations through observation of spermathecae [51].

The mean and median number of eggs laid per mosquito was higher among the field collected generation than the laboratory generations (Table 1), but this could only be tested statistically with individually maintained specimens. Our respective mean (14.3) and median (13) number of eggs per ovipositing field mosquito (Table 2) was lower than reported by Bates [15] who produced 22 eggs per ovipositing female. Mondet [16] produced 32.2 eggs per mosquito, but that was based on a sample of 10 specimens which had completed a single gonotrophic cycle. It is, therefore, worth noting that 153 of our 301 F_3_ eggs were laid by just seven mosquitoes within the space of two days (Dataset S1). Cages of free laying females, capable of passing through several gonotrophic cycles, will probably be required to maintain a colony given the small number of eggs produced per cycle. Better oviposition substrates, possibly mimicking tree holes [52], may improve egg yields. Furthermore, testing the effects of additional larval nutrients on reproductive output (fertility and fecundity) may be worthwhile, since the quality of larval nutrition can affect such adult phenotypes [53].

A total of 62.8% F_1_ generation eggs hatched during ≤10 immersion cycles, decreasing to 20.1% in the F_2_ generation. It has been suggested that a period of conditioning (partial drying) is required before *Hg. janthinomys* eggs will hatch [25,54], but also that repeated immersions during embryogenesis influence *Hg. leucocelaenus* hatch rates [55]. Season may also affect rates of eclosion among *Hg. janthinomys* and *Hg. leucocelaenus* [22,25,56]. Immediately initiating immersion cycles may mimic rainy season conditions resulting in increased eclosion, while a period of conditioning may simulate dry season conditions, but data are needed to clearly show this. Our installment hatching results are consistent with Alencar et al. [23] who showed negligible effects of immersions beyond the ninth cycle on *Hg. janthinomys* eclosion. Given that our work extended from the rainy season into the early dry season, there may have been a temporal effect on the percentage of eggs hatched. However, it is also likely that egg fertilization rates were lower among the F_2_ generation than among eggs laid by field mosquitoes.

Once hatched, rearing was relatively straightforward with 93.7% of F_2_ larvae surviving to adults. Poor survival among the F_1_ generation was associated with biofilm growth, a common problem when rearing [57,58], or with overcrowding resulting in the production of small adults. These were either unable to escape the surface tension of water or were difficult to use for mating. The first adults usually emerged within 13–16 days, corresponding reasonably well with estimated natural development times of 11–13 days for *Hg. janthinomys* [11]. We also documented an increase in *Hg. janthinomys* occurrence associated with increasing 7-day cumulative rainfall at a lag of 1 week in the field (i.e., an increase in adults 14 days after the onset of particularly heavy rainfall) [59]. In the laboratory, we found significant differences in development time at the larval stage between generations. The ad hoc addition of yeast was potentially confounding, but since food resources were plentiful, we believe the negative density-development relationship was accurate. The number of days until the first eggs were laid by field mosquitoes was similar to the 6.2 to 7.2 days reported by Bates [15], although the F_2_ generation took slightly longer to oviposit.

We reared larger mosquitoes in the insectary than we collected in the field, mainly to increase chances of survival and for ease of manipulation. It is known that the number of eggs produced by a mosquito is related to its size [60], so rearing larger mosquitoes should facilitate colony maintenance. However, mosquito size may also affect vector competence [61], which should be taken into consideration when planning *Hg. janthinomys* transmission experiments.

## 5. Conclusions

*Haemagogus janthinomys* is the major vector of YFV and thought to be a prominent vector of MAYV in the neotropics, but it is not known whether it can transmit DENV, ZIKV, or CHIKV. Our pilot attempts at laboratory maintenance should form the basis of future experimental studies. Our artificial blood feeding method will permit future vector competence studies involving this species.

## Figures and Tables

**Table 1:** Summary table showing progression from field collected mosquitoes through to F_3_ eggs for all mosquitoes included in the study.

Stage	N (%)	Summary
Field collected mosquitoes	418	
Blood fed	226 (54.1)	Range 29.1–90.5%
F_1_ eggs laid	2486	Mean 11 per ♀
F_1_ eggs hatched	1562 (62.8)	Range 51.4–87%
F_1_ reared to adult	1345 (86.1)	Range 19.2–100%
F_1_ ratio ♂:♀	638:707 (47.4:52.6)	
F_1_ ♀ copulated	501	
F_1_ ♀ in cages	445	
F_2_ eggs laid	944	Mean 2.1 per ♀
F_2_ eggs hatched	190 (20.1)	Range 3.1–41.4%
F_2_ reared to adult	178 (93.7)	Range 66.7–100%
F_2_ ratio ♂:♀	80:98 (44.9:55.1)	
F_2_ ♀ for blood feeding	92	
F_2_ ♀ blood fed	79 (85.9)	
F_2_ ♀ copulated	59 (73.4 *)	
F_2_ ♀ in Petri dishes	53	
F_3_ eggs	301	Mean 3.3 per ♀ **

* % blood fed mosquitoes copulated. ** 5.1 eggs per blood fed + copulated mosquito; 5.68 eggs per mosquito in Petri dish; 10.4 eggs per ovipositing mosquito.

**Table 2:** Summary table showing mean, median, and IQR for comparisons shown in Figure 5. Mean values are included to facilitate comparisons with previously published work [15,16]. ‘Gen.’ = Generation, ‘N =’ = number of individual mosquitoes.

Comparison	Gen.	N =	Mean	Median	IQR	Min.	Max.
Days until first eggs laid	Field	42	6.6	6	1	6	9
	F_2_	53	8.4	8	3	6	14
Eggs laid per mosquito for all individual mosquitoes in Petri dishes	Field	42	10.6	5	19.25	0	41
	F_2_	53	5.7	1	10	0	33
Eggs laid per mosquito for ovipositing mosquitoes only	Field	31	14.3	13	18	0	41
	F_2_	29	10.4	9	15.5	0	33

## Data Availability

The data presented in this study are available in the article and its supplementary material.

## Supplementary Materials

The following supporting information can be downloaded at: https://www.mdpi.com/article/10.3390/v15010045/s1, Figure S1: Generic cages, Figure S2: Oviposition in Petri dishes, Figure S3: Oviposition in cages, Video S1: Forced mating, Dataset S1: Complete dataset.
