# Supplementary material for: Towards the Laboratory Maintenance of Haemagogus janthinomys (Dyar, 1921), the Major Neotropical Vector of Sylvatic Yellow Fever
Source: Viruses. 2022 Dec 23;15(1):45. doi: 10.3390/v15010045 (PMC9861039; doi:10.3390/v15010045)

Supplementary figures

Towards the Laboratory Maintenance of *Haemagogus janthinomys* (Dyar, 1921), the Major Neotropical Vector of Sylvatic Yellow Fever

Adam Hendy, Nelson Ferreira Fé, Danielle Valério, Eduardo Hernandez-Acosta, Bárbara A. Chaves, Luís Felipe Alho da Silva, Rosa Amélia Gonçalves Santana, Andréia da Costa Paz, Matheus Mickael Mota Soares, Flamarion Prado Assunção, José Tenaçol Andes Jr., Chiara Andolina, Vera Margarete Scarpassa, Marcus Vinícius Guimarães de Lacerda, Kathryn A. Hanley and Nikos Vasilakis

Supplementary figures

Figure S1. Generic 15 x 15 cm plastic cage covered with fine mesh netting used for *Hg. janthinomys* adult maintenance and blood feeding. (**a**) lateral view; and (**b**) view from above with two cotton wool balls, one soaked with distilled water and the other a 10% sugar solution. Cotton wool was also used to plug a hole in the netting through which mosquitoes were added and removed.

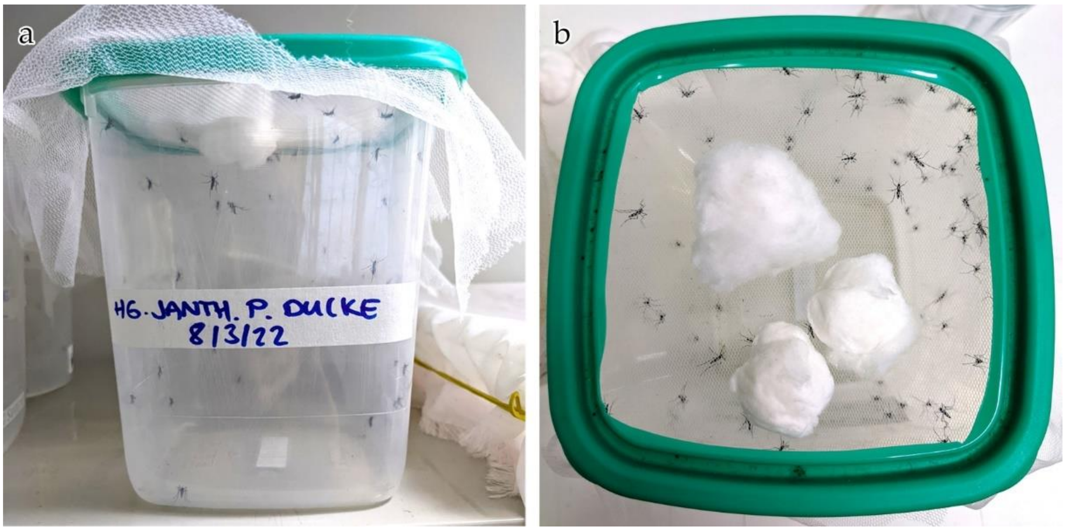

Supplementary figures

Figure S2. Oviposition methods for *Hg. janthinomys* maintained in Petri dishes. (**a**) anaesthetized female beneath a stereomicroscope with its wing removed; (**b**) anaesthetized females inside a Petri dish lined with 80 g filter paper placed above a damp cotton pad to maintain humidity; and (**c**) females attempting oviposition on filter paper at the edge of a Petri dish.

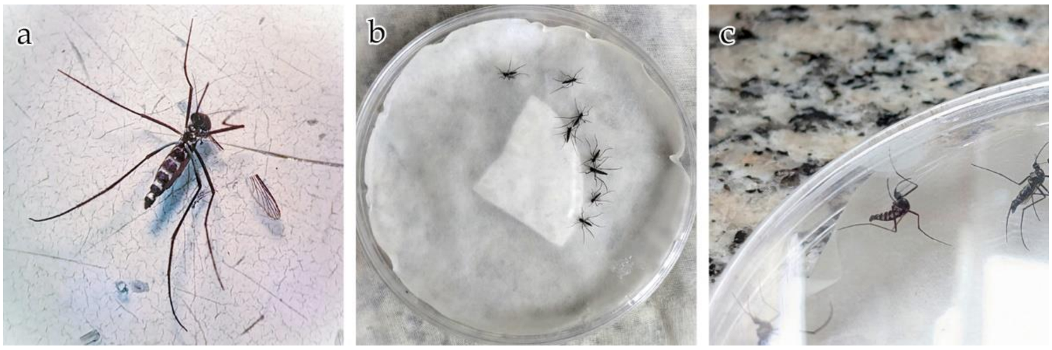

Supplementary figures

Figure S3. Oviposition methods for *Hg. janthinomys* maintained in cages. (**a**) 20 x 18 cm cage with F_1 generation females being offered blood and an oviposition substrate at the base of the cage; and (**b**) oviposition substrate with F_2 eggs laid on damp filter paper.

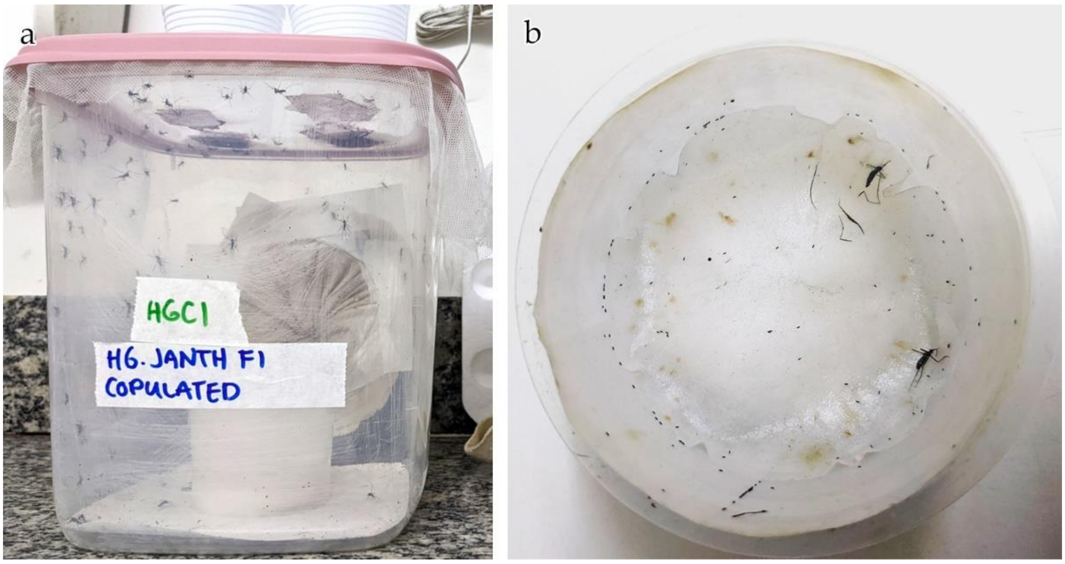

Supplement: Supplementary file 1 [file viruses-15-00045-s001.zip › Figures S1-S3.pdf]
